# Innate and Adaptive Lymphocytes in Non-Tuberculous Mycobacteria Lung Disease: A Review

**DOI:** 10.3389/fimmu.2022.927049

**Published:** 2022-06-28

**Authors:** Andrea Gramegna, Andrea Lombardi, Nicola I. Lorè, Francesco Amati, Ivan Barone, Cecilia Azzarà, Daniela Cirillo, Stefano Aliberti, Andrea Gori, Francesco Blasi

**Affiliations:** ^1^Department of Pathophysiology and Transplantation, University of Milan, Milan, Italy; ^2^Internal Medicine Department, Respiratory Unit and Cystic Fibrosis Adult Center, Fondazione IRCCS Ca’ Granda Ospedale Maggiore Policlinico, Milan, Italy; ^3^Internal Medicine Department, Infectious Diseases Unit, Fondazione IRCCS Ca’ Granda Ospedale Maggiore Policlinico, Milan, Italy; ^4^Emerging Bacterial Pathogens Unit, IRCCS San Raffaele Scientific Institute, Milan, Italy; ^5^IRCCS Humanitas Research Hospital, Respiratory Unit, Milan, Italy; ^6^Department of Biomedical Sciences, Humanitas University, Milan, Italy

**Keywords:** non-tuberculous mycobacteria, adaptive immunity, immune checkpoint inhibitors, immune exhaustion, immune dysfunction

## Abstract

Non-tuberculous mycobacteria (NTM) are ubiquitous environmental microorganisms capable of a wide range of infections that primarily involve the lymphatic system and the lower respiratory tract. In recent years, cases of lung infection sustained by NTM have been steadily increasing, due mainly to the ageing of the population with underlying lung disease, the enlargement of the cohort of patients undergoing immunosuppressive medications and the improvement in microbiologic diagnostic techniques. However, only a small proportion of individuals at risk ultimately develop the disease due to reasons that are not fully understood. A better understanding of the pathophysiology of NTM pulmonary disease is the key to the development of better diagnostic tools and therapeutic targets for anti-mycobacterial therapy. In this review, we cover the various types of interactions between NTM and lymphoid effectors of innate and adaptive immunity. We also give a brief look into the mechanism of immune exhaustion, a phenomenon of immune dysfunction originally reported for chronic viral infections and cancer, but recently also observed in the setting of mycobacterial diseases. We try to set the scene to postulate that a better knowledge of immune exhaustion can play a crucial role in establishing prognostic/predictive factors and enabling a broader investigation of immune-modulatory drugs in the experimental treatment of NTM pulmonary disease.

## Introduction

Non-tuberculous mycobacteria (NTM) are ubiquitous microorganisms that can cause severe infections, involving in 90% of cases the lungs (NTM-LD) (1). Cases of NTM-LD are increasing worldwide, with an incidence ranging from 8.6 to 17.7 cases per 100,000 person-year (2, 3). Once considered restricted to immunocompromised subjects, NTM infections are now identified also in otherwise healthy individuals (4).

Despite being environmental microorganisms, NTM cause disease only in a small subset of individuals at risk (5–7). The reasons behind this pleomorphic expression are not completely understood and have been linked to several factors. Among pathogen-related factors, some species of NTM have high pathogenicity, with *M. malmoense* and *M. kansasii* being almost invariably associated with disease, whereas others like *M. gordonae* do not cause pathology in the host (8). Host predisposing factors have been also identified, with female gender, history of cigarette smoking, and concomitant chronic lung disease as bronchiectasis, chronic obstructive pulmonary disease, silicosis or cystic fibrosis being among the constitutive or acquired elements associated with the development of NTM-LD (9). Also host factors related to the immune system, like deficiencies in CD4+ T cell function due to HIV infection, anti-tumour necrosis factor (TNF) therapy, and inherited deficiencies in the production or response to interferon-gamma (IFN-γ) have been associated with disseminated NTM infection (10).

The aim of this review is to provide a global overview of the cell-mediated immunity directed toward NTM infection, focusing on lymphoid cells belonging to both innate and adaptive immunity.

## The Role of Innate Lymphoid Cells

The innate lymphoid cell (ILC) family is composed of natural killer (NK) cells, ILC1, ILC2, ILC3, and lymphoid tissue-inducer cells. ILCs are only a small proportion of the total immune cells in the lung, but they have been found to promote lung homeostasis and are emerging as contributors to a variety of chronic lung diseases (11).

### NK Cells

Natural killer (NK) cells are a class of innate lymphoid cells exerting both cytotoxic and immune regulatory activities and are identified among the lymphocyte subset by the expression of the surface marker CD56 in the absence of CD3 (12–14). Their subset with low CD56 expression (NK^dim^) targets cells characterized by modified, downregulated, or absent host major histocompatibility complex class 1 molecules expression. Instead, the subset with high CD56 expression (NK^bright^) produces immunoregulatory cytokines like TNF-α or IFN-γ. The activity of NK cells is finely tuned by the balance of signals provided by activating (CD16, NKG2C/D, NKp30, NKp44) or inhibitory (NKG2A, TIGIT, KIR3DL1, KIR3DL2) receptors. NK cells, and especially NK^dim^, are well represented in the lung where they play major effector and immunoregulatory roles to ensure the self-integrity of the organ (15).

The role played by NK cells in the context of NTM-immunity is unclear, despite it is possible to speculate a relevant function related to their ability to kill cells infected by intracellular pathogens and their relevant production of IFN-γ, a key cytokine involved in the control of mycobacterial infections (16). In 1991, Bermudez and Young demonstrated in an *in-vitro* model that NK cells have an important role in inducing the killing in macrophages infected with *M. avium* complex, both directly and by stimulation of macrophages through TNF-α (16). Moreover, in a mouse-model, Feng et al. showed that the depletion of NK cells through anti-NK antibodies leads to uncontrolled multiplication of *M. avium* complex, suggesting that NK cells are crucial for the control of the infection and their absence cannot easily be abrogated by the immune system (17). Similarly, Lai et al. observed that NK cell depletion is associated with increase in bacterial load and mortality in a mouse model of NTM-LD due to *M. kansasi*. NK cell depletion exacerbated NTM-induced pathogenesis by reducing macrophage phagocytosis, dendritic cell development, cytokine production, and lung granuloma formation. Similar pathological phenomena were observed in IFN-γ-deficient (IFN-γ^-^/^-^) mice following NTM infection and the adoptive transfer of wild-type NK cells into IFN-γ^-^/^-^mice considerably reduced NTM pathogenesis. Furthermore, the injection of recombinant IFN-γ prevented NTM-induced pathogenesis in IFN-γ^-^/^-^mice, overall suggesting that IFN-γ production by NK cells activates and shapes innate and adaptive immune responses against NTM (18). On this scenario, IFN-γ production appears as the most important contribution of NK cells against NTM infection. In a mouse model of severe combined immunodeficiency, the administration of antibodies able to abrogate NK-mediated cytolysis did not affect *M. avium* infection, whereas the neutralization of IFN-γ led to a reduction in macrophage activation and subsequent exacerbation of mycobacterial growth (19).

Overall, NK cells appear to be important players in the immune response against NTM in the lung, especially through the production of the immunomodulatory cytokine IFN-γ. The understanding of their role is still incomplete and newer and more comprehensive investigations are needed.

### Innate Lymphoid Cells

Innate lymphoid cells (ILCs) are a group of innate immune lymphocytes, subclassified into three main classes originating from a common innate lymphoid progenitor cell (20).

Type 1 ILCs (ILC1) mediate a type 1 immune response through the production of IFN-γ and TNF-α (21). Although found in most human tissues, ILC1 are predominant in salivary glands, liver and the gastrointestinal tract, where they are supposed to activate against tumour cells and intracellular pathogens (22). Type 2 ILCs (ILC2) mediate type 2 immunity by the production of IL-4, IL-5 and IL-13 and have been associated with airway hyperactivity and allergic diseases (23). Type 3 ILCs (ILC3) are proposed to be equivalent to Th17 lymphocytes due to their production of IL-22 and/or IL-17 cytokines and are mainly involved in the gut microbiota-host homeostasis (24).

Experimental data have shown an involvement of ILCs in *M. tuberculosis* (*Mtb*) infection with a possible role in determining the outcome of the disease.

In a recent study, Corran et al. investigated local activation of lung resident ILCs in a murine model of *Mtb* infection. They reported that *Mtb* had a profound impact on ILC phenotype and induced a differentiation of ILC precursors toward an IFN-γ-producing subset (ILC1-like cells) (25).

Ardain et al. showed both depletion of ILC subgroups, especially ILC1 and ILC2, in the peripheral blood of patients with ongoing tuberculosis and their restoration after clearance of *Mtb*. The authors speculated that circulating ILCs are not lost from the blood due to cell death, but they migrate to the site of infection. In confirmation of this hypothesis, the authors demonstrated resident ILCs in lung biopsies of *Mtb*-infected participants using established markers. Consistently with these findings, ILC3 accumulation in the lungs of animal models was postulated as instrumental to massive macrophage recruitment in response to mycobacterial infection and mice lacking ILC3 showed reduced macrophage accumulation and poor infection control (26, 27).

Regarding NTM, no data are currently available about the role of ILC, even though is possible to postulate a lung recruitment and activation of them in the early stages of NTM-LD.

## Unconventional T Cells

The family of unconventional T cells is composed of various subsets of innate-like effectors, which play a role in the protection against infectious non-self by mounting rapid immune responses and showing immunoregulatory capabilities. In particular, the subsets of unconventional T cells that have been better characterized are natural killer T (NKT) cells, mucosal-associated invariant T (MAIT) cells, and γδ T cells.

These subsets are defined by the expression of highly evolutionarily conserved semi-invariant αβ or γδ T-cell Receptors (TCR), which allow unconventional T cells to sense lipidic antigens, small molecules derived from microbial metabolism and other non-polymorphic molecules (28, 29). Furthermore, each unconventional TCR is able to recognize multiple antigenic specificities, aiming predominantly at pattern recognition of structures that are present across microbic species and are highly conserved (30). All unconventional T cells develop from common T cells precursors in the thymus, where they acquire the capability of moving to barrier tissues (28). Here, unconventional T cells can both mount immune responses based on the release of massive amounts of cytokines and stimulate the differentiation of CD4+ or CD8+ T-cell lineages (31).

### Natural Killer T Cells

Natural killer T (NKT) cells constitute a subset of unconventional αβ T lymphocytes (32). Their peculiarity consists of the expression of a highly evolutionarily conserved αβ TCR, which is activated by the stimulation of lipidic and glycol-lipidic antigens presented by antigen-presenting cells (APC) through major histocompatibility complex (MHC) class I-like CD1d molecules (29).

Based on differences in the TCR structure, NKT cells are classified into two subsets. The NKT1 subset (also referred to as iNKT as in invariant NKT cells) is characterized by the expression of the semi-invariant TCR, which exposes Vα14-Jα18 chains paired with Vβ11 sequences in humans (31, 33). The NKT2 subset, on the other hand, express a broader TCR repertoire (34).

During their maturation, NKT cells undergo extensive expansion and acquire the capability of migrating towards barrier-like tissues, where it was demonstrated the majority of peripheral NKT cells reside (33, 35, 36). In contrast to NKT cells, conventional T cells respond to the stimulation by MHC class I and II molecules, which are highly polymorphic and, by random rearrangements of genes coding for TCRα and TCRβ chains, can express an impressing diversity, theoretically recognizing peptide antigens from any given microbe. Conventional T cells are capable of exerting this function at the expense of any antigen-specific clone being rare and therefore require clonal expansion before being able to generate a sufficiently large population of effector T cells (31).

On the other hand, NKT cells show a less diverse TCR, which responds to molecular structures that are shared by different pathogens, rather than to specific peptide antigens (37). As already mentioned, NKT cells already went through expansion when egressing the thymus and are then able to express a rapid “first-line” immune response to pathogens encountered in barrier tissues (33).

About the role of NKT cells in the defence against NTM no data is available. Nonetheless, evidence gathered studying *Mtb* can be probably exported due to the similarities between these pathogens. As stated above NKT cells have the ability to recognize lipidic and glycol-lipidic antigens presented by APC. For example, diacyl-glycerols (DAGs) and phosphatidyl-inositol-mannoside (PIM4), expressed on the surface of both *Mtb* and NTM, are known to be important NKT cell bacterial antigens (35). *Mtb* and NTM share many of these molecules in their structure. An example is lipoarabinomannan, a major structural component of cell membrane of mycobacteria as well as an important virulence factor for *Mtb* (38). Responses against antigens of this class have been described for NKT cells (39–43).

Clinical features have been linked with these responses, with the presence of NKT cells both in peripheral blood and bronchoalveolar lavage fluid, strongly activated by lipoarabinomannan, being associated with the ability to control *Mtb* infection in an *ex-vivo* study (44). Moreover, the expression of CD1d, the MHC molecules required for the presentation of antigens to NKT cells, has been described on macrophages and dendritic cells from *Mtb* granuloma, suggesting a crucial role of these lipid-specific T cells in the shaping of the immune response toward this pathogen (45). NKT cells express their effector functions by massive release of cytokines, particularly IL-4 and IFN-γ, the latter of which was described as central in the regulation of cell-mediated response toward NTM (35, 46). In addition to enhancing macrophage intracellular killing capabilities, IFN-γ and IL-12 were found to be important in stimulating the recruitment of CD8+ T cells and the differentiation of CD4+ T cells into T-helper 1 cells. These findings are consistent with the role of NKT in bridging innate and adaptative immune responses toward NTM (35).

### Mucosal-Associated Invariant T Cells

Mucosal-associated invariant T (MAIT) cells are a subpopulation of innate-like T lymphocytes, which express an invariant TCRα chain and display effector-like characteristics in the immune response to several pathogens. More specifically, MAIT cells are defined by the expression of the iVα7.2- Jα33 TCRα chain, coupled with a limited number of Vβ chains (e.g. Vβ2 or Vβ13) (47–49). MAIT cells recognize vitamin B-based antigens presented by the non-polymorphic MHC class I related-1 molecule (MR1).

The first *in vivo* evidence of the antimicrobial role of MAIT cells came by Le Bourhis et al., which showed that MR1-deficient mice were exposed to increased bacterial burden in comparison to mice expressing wild type levels of MAIT cells when injected intra-peritoneally with *E. coli* and *M. abscessus* (50). It is worthy to notice that the stimulation of MAIT cells might also be independent of the interaction between MR1 and TCR and, in this case, is mediated by cytokines. The MR1-independent pathway of MAIT cells activation was found to be relevant in the MAIT cells-mediated response against viruses and NTM (51). To demonstrate that, in 2012, Chua co-cultured MAIT cells and BCG-infected macrophages in the presence of anti-MR1 antibodies and still observed MAIT cells-mediated response, resulting in the production of IFN-γ and IL17-A, and the consequent enhancement of intracellular killing of mycobacteria (52). Furthermore, in 2019 Suliman et al. confirmed that MAIT cells activation against BCG was largely mediated by both IL-12 and IL-18, and to a much lesser extent by MR1-TCR triggering (53).

Interestingly, lower frequencies of peripheral blood MAIT cells have been observed in patients with active *M. avium* complex pulmonary infection, and particularly with cavitary disease, when compared to healthy donors (54). These lower frequencies of peripheral blood MAIT cells were observed also during NTM-LD due to other mycobacterial species and were significantly correlated with several clinical and biochemical variables like sputum AFB positivity, extent of disease, haemoglobin levels, lymphocyte counts, CRP and ESR levels (48). The same study also described a reduced production of IFN-γ by MAIT cells from NTM patients, underlying the importance of this cytokine in the pathogenesis of NTM-LD. Finally, an exciting therapeutic role for MAIT cells has been suggested by Wakao et al. Employing a Sendai virus harbouring standard reprogramming factors, they were able to reprogram MAIT cells in to induced pluripotent stem cell (iPSC). Under T cell-permissive conditions, these iPSCs efficiently re-differentiate into MAIT-like lymphocytes that, after incubation with *E. coli*-fed monocytes, show enhanced production of a broad range of cytokines, including IFN-γ, (of which about a 20-fold increase was observed), IL-2, IL-17, IL-10 and TNF-α. Following adoptive transfer into immunocompromised mice, these derived MAIT cells migrated to the bone marrow, liver, spleen, and intestine and protected the recipient against *Mycobacterium abscessus* (55).

### Gamma-Delta T Cells

The family of γδ T lymphocytes constitute a subset of unconventional T cells that express on their surface a peculiar invariant or semi-invariant TCR heterodimer, composed of one Vγ9 and one Vδ2 glycoproteic chains (56). This family of T cells is thought to be less frequent than canonical αβ T cells in peripheral blood, while taking their place preferentially in peripheral mucosal tissues, the skin and presumably the lung and the peritoneal cavity (57).

Here γδ T cells express effector capabilities by rapidly producing an array of cytokines, primarily IL-17. In these settings, IL-17 production is initiated by γδ T cells after recognition of poorly defined polymorphic structures, *via* their semi-invariant TCR, without the need for presentation by APCs. Some authors also demonstrated that stimulation with inflammatory cytokines, mainly IL-23 and IL-1 produced by bystander dendritic cells, is possible and does not require TCR engagement (58, 59). By doing so γδ T cells represent a crucial innate source of immunoregulatory cytokines in first-line response to pathogens. In this regard, it is ultimately not surprising that γδ responses to mycobacteria were described as early as 1989 (60). Of note, it was also reported more recently the possibility for γδ T cells to differentiate in secondary lymphoid organs to produce IL-17 after antigen-specific engagement of their TCR. This was described by Chien et al. in 2012 as an “inducible” IL-17 production by γδ T cells, as opposed to the “innate” IL-17 production we mentioned above (61). In addition, is well known the opportunity for γδ T cells to stimulate CD4+ Th1-dependent immunity: this is consistent with the notion of γδ T cells being one of the most complex components of the innate immune system, capable also to play a role in adaptive immunity (56).

The role of γδ T cells in NTM-LD is not clearly defined, due to a paucity of studies assessing their characteristics and dynamics. As stated above, γδ T cells are early recruited at the site of infection and produce cytokines that will shape the response against the pathogen. This was confirmed in a calf-model of *Mycobacterium avium* subspecies *paratuberculosis* infection, where γδ T cells were early recruited at the site of infection, their prevalence decreased during the infection, and produced significant amount of IFN-γ (62). In addition, in a ruminant model, early *Mycobacterium bovis* infection led to an increase in numbers of activated γδ T cells in both the lung and blood, without an apparent expansion of total circulating γδ T cells (63). In humans, γδ T cells are supposed to be the immune cytotypes devoted to respond mainly to fragments of mycobacteria. Indeed, in a study that compared the proliferation of distinct human T cell subsets in response to live, killed or soluble extracts of *Mtb* and *M. avium* complex, when soluble bacterial extracts were used as stimulators, a preferential proliferation of γδ T cells, expressing predominantly Vγ9+ and Vδ2+ T cell receptor chains, was recorded (64). Finally, *ex-vivo* studies performed among HIV patients with disseminated *M. avium* complex infections highlighted an expansion of γδ T cells in the peripheral blood (65, 66). These results must be interpreted with caution, considering that they were obtained in severely immunocompromised patients where the expansion of γδ T cells population can represent a deficiency in other cellular subsets.

## Conventional T Cells and Immune Exhaustion

A crucial branch of adaptive immunity involved in the control of mycobacterial infections is represented by T lymphocytes, both CD8+ and CD4+. In the advanced stages of the human immunodeficiency virus (HIV) infection, severe and disseminated mycobacterial infections are a common manifestation. This is related to the complex impairment of the immune system due to HIV infection, involving CD4 T cells and myeloid cells such as macrophages. Particularly, the loss of appropriate and efficient CD4 T-cell responses associated with type 1 cytokine secretion (IFN-γ and TNF-α), is essential to controlling these intracellular pathogens (67). The importance of these pathways is corroborated by the association between defects in the IFN-γ pathway, due to mutations involving the IFN receptor, and NTM infection (68, 69).

Recently, another immune dysfunction regarding T lymphocytes has been associated with NTM infection. This condition is called immune exhaustion (IE) and it is defined by poor effector function (e.g. reduced IFN-γ production) and a sustained expression of inhibitory receptors (cytotoxic T-lymphocyte-associated protein 4 [CTLA-4], programmed cell death protein 1 [PD-1], and T-cell immuno- globulin domain and mucin domain 3 [TIM-3]), on the surface of T lymphocytes, which prevents optimal control of infections and tumours (70). IE is induced by several mechanisms like cell-to-cell signals including prolonged TCR engagement and co-stimulatory and/or co-inhibitory signals, soluble factors such as excessive levels of inflammatory cytokines and suppressive cytokines, and tissue and microenvironmental influences driven by changes in the expression levels of chemokine receptors, adhesion molecules and nutrient receptors (71). IE has been initially described in the setting of chronic viral infection, but it has been subsequently observed also in several tumours (72). Currently, innovative drugs called immune checkpoint inhibitors, able to re-establish a correct immune response toward the neoplastic antigen, are widely employed and are reshaping the landscape of cancer treatment with outstanding results (73).

A growing bulk of evidence is accumulating, highlighting how IE features can be identified in NTM-infected patients. Shu et al. showed that patients with *M. avium* complex-induced lung disease (MAC-LD) had a weak *in vitro* peripheral blood mononuclear cells (PBMC) response to NTM antigens, assessed in terms of IFN-γ production, and a higher expression of PD-1 and apoptosis markers on these cells. Interestingly, during antimycobacterial treatment, TNF-α and IFN-γ production by PBMC increased, supporting the notion of a relevant impact on T cell function by decreasing antigenic burden. Moreover, the partial block of PD-1 and the PD ligand with antagonizing antibodies significantly increased the cytokine production of IFN-γ and decreased the expression of apoptosis markers on T lymphocytes, highlighting how immune checkpoint pathways modulate T cell responses during MAC-LD (74). These results have been corroborated by Han et al., who highlighted an increase in PD-1, CTLA-4, and TIM-3 expression on CD4+ T cells in MAC-LD individuals after NTM-antigens stimulation (75) and Wang et al., who showed that in patients with NTM-LD, TIM-3 expression increased over CD4+ and CD8+ T cells and correlated with cell apoptosis and a reduction in specific cytokine production (IL-2, INF-γ, TNF-α) (76). In addition, Shu et al. found a higher number of PD-1+CD4+ lymphocytes and myeloid-derived suppressor cells in MAC-LD patients compared to controls and associated these alterations with a higher burden of mycobacterial bacilli, again stressing the importance of antigenic burden in the development of IE features (77). Finally, Lutzky et al. suggested that different phenotypes of IE can be present in patients with NTM-LD, related to the host factors associated with the development of the infection. Indeed, comparing cystic fibrosis patients and elderly patients with *Mycobacterium abscessus* complex infection to healthy controls, they highlighted a unique surface T cell phenotype with a marked global deficiency in TNFα production in the first group and a different phenotype expressing exhaustion markers and dysregulation in type 1 cytokine release in the latter (78). **Table 1** provides a summary of the studies investigating conventional T cell immune exhaustion in NTM-LD.

**Table 1:** Summary of studies investigating conventional T cell immune exhaustion in NTM-LD.

Study ID	Patients included	NTM species	Phenotypic properties	Functional properties
Shu et al. Scientific Reports, 2017	80 participants: - 50 MAC-LD - 30 HC	MAC	Lymphocytes of patients with MAC-LD have higher PD-1, PD-L1 and apoptosis markers expressions than those of healthy controls.	Patients with MAC-LD had lower TNF-α and IFN-γ responses compared to HC in PBMC stimulation assays with MAC bacilli. MAC therapy improved the secretion of TNF-α and IFN-γ. Partially blocking PD-1 and the PD-L1 with antagonizing antibodies significantly increased the cytokine production of IFN-γ of MAC-LD.
Han et al. Journal of Clinical Medicine, 2020	91 participants: - 71 MAC-LD - 20 HC	MAC	In patients with MAC-LD, CD4+ T cells and CD4+IL-17+ T cells frequencies decreased and CD4+IL-4+ T cells and CD4+CD25+Foxp3+ T cells (Tregs) increased after MAC stimulation compared to HC PBMC. MAC-LD patients have an increased PD-1, CTLA-4, and TIM-3-expression on T cells in response to MAC-stimulation compared to HC in PBMC.	Patients with MAC-LD had lower IFN-γ, IL-17A, IL-10 production compared to HC in PBMC stimulation assays with MAC bacilli.
Wang et al. Frontiers in Immunology, 2021	93 participants: - 47 MAC-LD - 46 HC	MAC	Patients with MAC-LD have a higher TIM3+ expression on CD4+ and CD8+ T cells compared to HC.	Patients with MAC-LD had higher cell apoptosis and specific cytokine attenuation (↓ secretion of IL-2, TNF-α, IFN-γ) compared to HC in PBMC stimulation assays MAC therapy (11 patients) decreased the TIM3+ expression on CD4+T and CD8+T cells after 2 months.
Shu C.C. et al. Journal of Clinical Medicine, 2019	96 participants: - 46 MAC-LD - 23 MABS-LD - 27 HC	MAC MABS	In the MAC-LD group, frequencies of PD-1+CD4+ T cell were higher than in HC and in MAB-LD patients. In the MAC-LD cohort were identified MAC subspecies: patients with *M. intracellulare* and *M. avium* have higher expression of PD-1 on CD4+ T cell compared to other subspecies (*M. chimera* and *M.timonense*) in the same cohort. No intergroup differences regarding CTLA-4+CD4+ and Treg cells frequencies. The proportion of MDSCs was higher in the MAC-LD and MAB-LD groups than among HC.	No functional properties are available: this article analysed association of phenotypic properties with clinical features and radiographic outcomes.
Lutzky VP et al. Frontiers in immunology, 2018	34 participants: - cohort of 24 CF patients: (CFAct) 7 with active pulmonary MABS infection; (CFPast) 8 with previous diagnosis of MABS infection; (CFControl) 9 with no history of or current NTM infection - cohort of 10 elderly patients: who had active or past NTM infection; HC	MABS	- Comparison of Tregs (CD4+ CD25+ FOXP3+): → higher percentages in CFAct and CFPast groups compared to the CFControl → in elderly cohort were higher in NTM patients compared to healthy controls. - CD25+, CTLA-4, and PD-1 on CD4+ T cells revealed a common fingerprint in CFAct and CFPast groups which was distinct from the CFControl group - no difference between CF patients with active or past NTM-PD and HC in terms of T cell fingerprint	- Post-mitogen stimulation TNFα-producing CD4+ T cells were significantly lower in both CFAct and CFPast groups compared to the CFControl group - Increased IFNγ secretion was seen in both CD4+ and CD8+ T cells in the CF NTM cohort compared to healthy controls; in the elderly NTM cohort, there was no significant increase in IFNγ-secreting cells in both CD4+ or CD8+ T cells.

NTM, non-tuberculous mycobacteria; MAC, M. avium complex; MAC-LD, MAC - lung disease; MABS, M. abscessus complex; HC, healthy controls; PBMC, peripheral blood mononuclear cells; MDSCs, Myeloid-derived suppressor cells; CTLA-4, cytotoxic T-lymphocyte-associated protein 4; PD-1, programmed cell death protein 1; TIM-3, T-cell immunoglobulin domain and mucin domain 3; CF, cystic fibrosis.

An indirect additional element suggesting how IE is involved in NTM pathophysiology derives from several case reports describing patients who experienced recrudescence of NTM-LD while receiving cancer treatment with immune checkpoint inhibitors (79, 80). The hypothesis is that the immune system of these patients, under the impact of immune checkpoint inhibitors, mounted a vigorous response against a pre-existing but clinically silent NTM infection, leading to clinical manifestations and disease. This also suggests that the blockade of immune checkpoints might be carefully evaluated to avoid immunopathology in the host, deriving from a re-established optimal T cell function. This note of caution is supported by the recent data provided by Kaufmann et al., who showed that in a macaque animal model of *Mtb* infection the administration of anti-PD1 antibodies led to worse disease and higher granuloma bacterial loads compared to isotype control-treated monkeys. PD-1-mediated co-inhibition seems required for control of *Mtb* infection in macaques, perhaps due to its role in dampening detrimental inflammation as well as allowing for normal CD4 T cell responses (81).

Taken together, these data suggest that lymphocytes IE is recognizable during NTM-LD and that it has a crucial, yet not completely understood, role in the disease. Future research should address its possible employment as prognostic/predictive factor and the coadministration of immune checkpoint inhibitors with standard antibacterial therapy in order to achieve quickly and in a more stable manner the control of the disease (82).

## Conclusions

A complete understanding of the lymphoid, cell-mediated, immune responses toward NTM causing pulmonary disease is far to be completed. Only a scarce number of studies have investigated the different cytotypes during this condition, and a large bulk of data are translated from study performed in animal models or involving the “cousin” pathogen Mtb. The lack of information about the interaction between NTM and the immune system actors analysed in our review probably hampers our capacity of understanding the pathophysiology leading to NTM-LD development and also the possibility of developing newer diagnostic and therapeutic tools.

The most promising area in this field appears T cell immune exhaustion. Measuring the expression of immune exhaustion markers on the surface of NTM-specific T cells or their production of immunomodulatory cytokines should be investigated as an instrument to quantify objectively the immune dysfunction of NTM-LD patients and to follow the response to the antimycobacterial therapy. Moreover, also the administration of immune checkpoint inhibitors, able to restore the function of exhausted T cells, should be investigated as adjuvant therapeutic approach during the treatment of NTM-LD. To avoid detrimental immunopathology deriving from a re-established optimal T cell response, it will be probably necessary to reduce the antigenic burden through canonical antimycobacterial treatment before the administration of immune checkpoint inhibitors.

The progress made by immunology in the last years, with the widespread availability of sophisticated instruments able to dissect the immune responses at the single-cell level, will probably help in understanding how we deal with NTM and why only a discrete subgroup of individuals develop the disease, a task indispensable considering the growing clinical relevance of NTM-LD and the paucity of diagnostic and therapeutic instruments.

## Author Contributions

Conceptualization: AGr and AL. Data collection: IB and CA. Methodology: AGo, AL, AGr, and FB. Writing - original draft: IB, CA, and AL. Writing - review and editing: AGr, AL, IB, and CA. All authors read and approved the final manuscript.

## Conflict of Interest

The authors declare that the research was conducted in the absence of any commercial or financial relationships that could be construed as a potential conflict of interest.

## Publisher’s Note

All claims expressed in this article are solely those of the authors and do not necessarily represent those of their affiliated organizations, or those of the publisher, the editors and the reviewers. Any product that may be evaluated in this article, or claim that may be made by its manufacturer, is not guaranteed or endorsed by the publisher.
